# The impact of climate change on extensive and intensive livestock production systems

**DOI:** 10.1093/af/vfy028

**Published:** 2018-10-26

**Authors:** Jean M Rust

**Affiliations:** Döhne Agricultural Development Institute, Stutterheim, South Africa

**Keywords:** adaptive changes, animal production, environment, livestock producers, product demand

**Implications**

- Extensive and intensive livestock production both contributes and is affected by climate change.
- There is considerable pressure on livestock production to deliver, under changing environmental conditions, on an ever-increasing demand for protein in human diets.
- Delivery on the increase in demand will not be possible without drastic changes to both extensive and intensive production.
- These adaptations/changes should contain mitigation components, which will enable the industry to deliver on the production and environmental demands; however, these changes will come at a monetary cost to producers and consumers.

## Introduction

The majority of animal scientists and livestock producers are fully aware and accept that the livestock production sector contributes to factors causing climate change and that in turn livestock production will also be affected by climate change. These effects will be both direct and indirect (Houghton et al., 2001). The impact of climate change on animal production has been categorized as the following: 1) availability of feed in the form of grain, 2) pasture and forage crop production and quality, 3) health, growth, and reproduction, and 4) diseases and the spread thereof (Rotter and Van de Geijn, 1999). In this article, the potential impact of projected climate change on the different livestock production systems (extensive and intensive) will be discussed in general with emphasis on the adaptation aspect. It is, however, important to put the livestock production sector into perspective before speculating on potential future changes.

What do we mean when we talk about livestock production and what is the definition of livestock? It is defined as domesticated animals raised in an agricultural production system with the aim of producing food, fiber, and labor. Sometimes, reference is only made to ruminants, such as cattle, sheep, and goats but this definition should include all livestock which fits the original description, including poultry, pigs, and so on.

Over time the livestock sector has increased in size and relative production output, especially in intensive animal farming practices (Muir, 2011). The increase in intensive beef cattle production in beef feedlots is due to the increasing global demand for protein (Millen et al., 2011; Costa Junior et al., 2012). In Brazil, Costa Junior et al. (2012) reports that the number of beef cattle fed in feedlots has more than doubled since 2012. Verge et al. (2008) ascribe this to the fact that this increase was driven by both population increases and the increased demand for higher rates of protein inclusion in human diets. A positive correlation exists between the expansion of beef cattle enterprises and those for the other species, where the same trend is observed. This increase has also been observed by the IPCC where an estimated 1.4-fold increase in numbers for cattle, buffalo, sheep, and goats, and a 1.6- and 3.7-fold increase for pigs and poultry, respectively, has taken place since 1970s (Smith et al., 2014).

**Figure 1.**
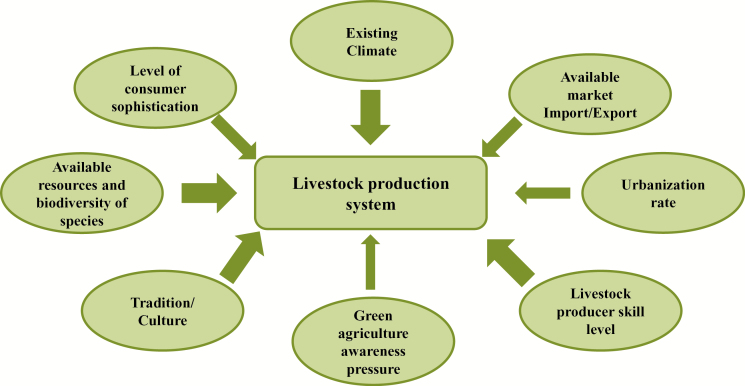
Main drivers of a specific livestock production system *(*weight of arrows indicate relative importance**).

Livestock systems, especially in developing countries, are extremely dynamic and various drivers of change can be identified. This includes increasing populations and incomes which are combining to drive considerable growth in demand for livestock products. This is projected to continue well into the future (Delgado et al., 1999), although at diminishing rates (Steinfeld et al., 2006). A second feature of the growing demand for livestock products is the shift in the location of production. An example of this is the rapid urbanization of (particularly monogastric) livestock production (the landless monogastric production system—LLM systems), followed in time by ruralization again. This second ruralization move is primarily in response to environmental drivers, meaning that after the initial urbanization, the pressures on resources and environmental pollution forces these production systems to less densely populated rural areas again. In addition to the factors associated with the “livestock revolution” (Delgado et al., 1999) and “livestock in geographic transition” (Steinfeld et al., 2006), other drivers may have far-reaching impacts on the livestock sector in the coming decades: the green agriculture movement (organic food, fair trade, etc.) and the increasing importance of fodder crops being grown for biofuel, for example. There may be considerable impacts of climate change on agricultural systems in the future, but it is clear that climate change is only one of several key drivers of change. Other factors such as population growth, globalization, urbanization, changing socioeconomic expectations, and cultural preferences, for example, may have a considerable impact on the system and on food security. The most important factors influencing a specific livestock production approach can be summarized in Figure 1.

Global livestock production is not uniform. There are differences in livestock production needs between developed and developing countries. These differences even exist within countries where certain areas may favor a certain approach to livestock production. Although both intensive and extensive production systems are practiced in both developed and developing countries, the trend is for production systems to be more intensive in the developed countries as compared with a more extensive approach in developing countries. Knowledge of the distribution of livestock resources can be applied in many ways, for example, in estimating production and off-take, the impacts on the environment, disease risk and impact, and the role that livestock plays in people’s livelihoods (FAO, 2007; Robinson et al., 2007). Livestock in different contexts serve quite different functions, play different roles in people’s livelihoods, vary in herd structure and breed composition, and are subjected to very different husbandry systems (Robinson et al., 2011).

These differences are mainly driven by internal factors, such as economic development, resource availability, population dynamics and rate of urbanization, culture, etc. (Figure 1).

## What Is the Role and Importance of Livestock Production?

It is estimated that grasslands cover approximately 30% of the earth’s ice-free land surface and about 70% of its agricultural lands (White et al., 2000; WRI, 2000; FAO, 2005). Livestock, and more specifically ruminants, are still the most effective organisms to convert grass into protein. An estimated 1 billion people depend on livestock, and 70% of the 880 million rural poor are to some extent dependent on livestock for their livelihoods (World Bank, 2007). Livestock production is practiced on two-thirds of global drylands (Clay, 2004). Extensive pastoralism occurs on 25% of global land surface and supports around 200 million subsistence pastoral households (Nori et al., 2005). In Africa, 40% of the land is dedicated to pastoralism (IRIN, 2007) and 70% of the population relies on dry and subhumid lands for their daily livelihoods.

**Figure 2.**
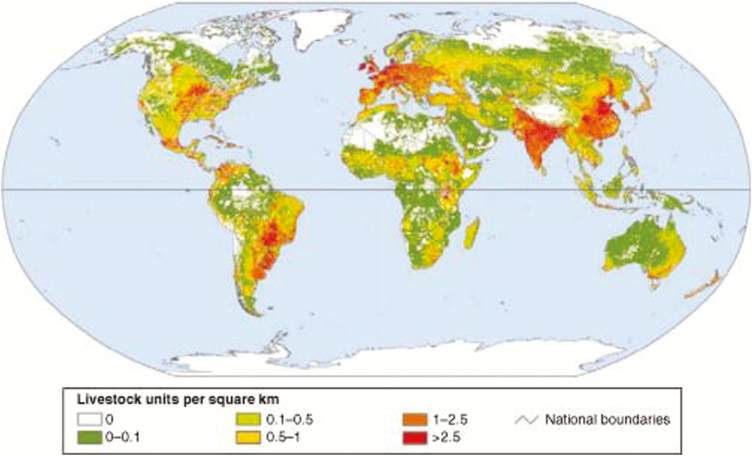
Global density of livestock (units per square kilometer) (FAO, 2006).

Twenty-three percent of the world’s poor (nearly 300 million people) are located in sub-Saharan Africa, and about 60% of these depend on livestock for some part of their livelihoods (Thornton et al., 2002). In sub-Saharan Africa alone, 25 million pastoralists and 240 million agro-pastoralists depend on livestock as their primary source of income (IFPRI and ILRI, 2000). Figure 2 illustrates the global density of livestock.

The type of production systems utilized shows more or less the same pattern, with intensive systems more dominant in the high-density regions and vice versa in the low-density regions.

Livestock products are the main outputs of natural and planted pastures and continue to be the fastest growing agricultural subsector globally. In some developing countries, the livestock sector accounts for 50–80% of GDP (World Bank, 2007). This gives us some indication on how important livestock and livestock production is for the world population and the global economy.

Livestock production is estimated to be responsible for 37% of global anthropogenic (originating from human activity) methane (CH_4_) emissions and 65% of anthropogenic nitrous oxide (N_2_O) emissions (FAO/LEAD, 2006). Methane from enteric fermentation in livestock is reported to be 85.63 million tonnes while the contribution from manure is estimated to be 18 million tonnes annually (FAO/LEAD, 2006). Of the total methane emissions from enteric fermentation, grazing systems contribute some 35% compared with 64% for mixed farming systems (FAO/LEAD, 2006). This illustrates the “catch twenty two” situation we are in—we are fully aware how detrimental livestock is to the environment but we can’t do without them.

## What Are the Different Livestock Production Systems?

**Figure 3.**
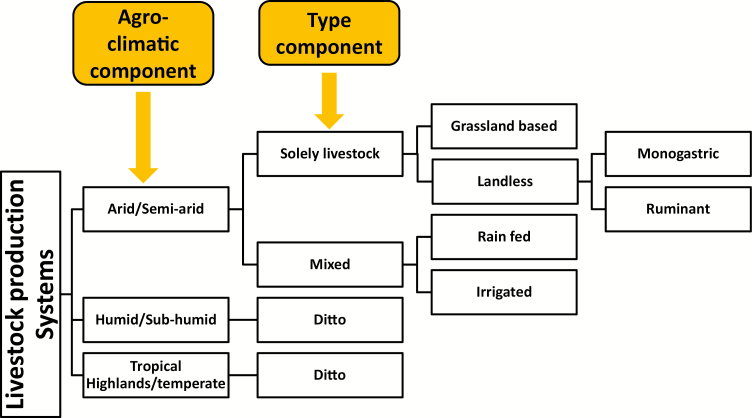
Schematic livestock production classification system.

Livestock production is categorized according to the classification system devised by Seré and Steinfeld (1996; Table 1). This classification system consists of two main criteria, namely agro-climatic and type. Illustration of the components of livestock production systems is shown in Figure 3.

**Table 1.** Livestock production systems simplified and coded (Seré and Steinfeld, 1996)

Generic	Specific	Systems
LG (livestock only)	LGA	Livestock only/arid/semi-arid
	LGH	Livestock only/humid/subhumid
	LGT	Livestock only/highlands temperate
MR (mixed rainfed)	MRA	Mixed rainfed crops/livestock/arid/semi-arid
	MRH	Mixed rainfed crops/livestock/humid/subhumid
	MRT	Mixed rainfed crops/livestock/temperate
MI (mixed irrigated)	MIA	Mixed irrigated crops/livestock/arid/semi-arid
	MIH	Mixed irrigated crops/livestock/humid/subhumid
	MIT	Mixed irrigated crops/livestock/temperate
LL (landless)	LLM	Landless monogastric
	LLR	Landless ruminant

**The following definitions apply:**

- Agro-climatic criteria—based on the length of growth period (**LGP**). Growth period is defined as the period in days during the year where the available rain fed moisture in the soil is greater than 50% of potential evapotranspiration. Excluded are periods of a mean temp of less than 5 °C.
- Type criteria—whether it is a livestock only system or mixed farming system where a crop production element is included.
  - Arid/semi-arid—LGP of less or equal to 180 days.
  - Humid/subhumid—LGP of more than 180 but less or equal to 270 days.
  - Tropical highlands/temperate—LGP of more than 270 days and month or more with sea level corrected temp of below 5 °C, during growth period the mean temperature is between 5 and 20 °C.
  - Solely livestock—where 90% of dry matter comes from rangelands, pastures, annual forages, and purchased feeds and less than 10% of production comes from nonlivestock activities.
  - Mixed system—more than 10% of dry matter fed to animals comes from crop by-products, stubble, or more than 10% of total value of production comes from nonlivestock farming activities.
  - Grassland-based systems—more than 10% of dry matter is produced on the farm.
  - Landless system—less than 10% of dry matter is produced on farm.
  - Rainfed mixed farming systems—more than 90% of nonlivestock farm production is from rainfed land use.
  - Irrigated mixed farming systems—more than 10% of value from nonlivestock production comes from irrigated land use.
  - Monogastic—value of pig/poultry production is more than ruminant.
  - Ruminant—ruminant production is higher than pig/poultry.

For the purpose of this article, these systems will only be discussed under the two main generic criteria, namely extensive and intensive systems.

## How Will Extensive Systems Adapt/Change under a Predicted Climate Change Scenario?

**Figure 4.**
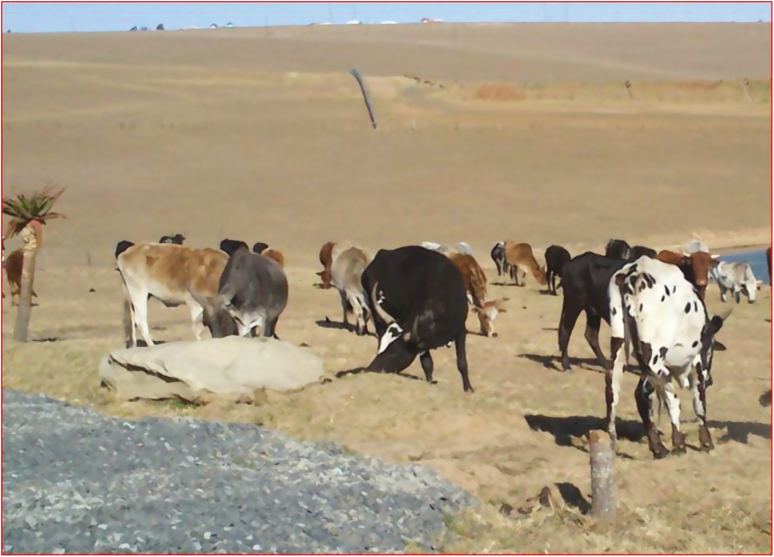
An extensive livestock production scenario with a high environmental cost and not effectively contributing to overall production.

It is suggested that extensive livestock production systems will come under increased pressure with predicted climate change scenarios (Figure 4). The causative factors are in the introduction. The following are predicted adaptive changes to be made to cope with a changed climatic scenario and to satisfy increased product demand:

- The net effect will in most probability be a slight decrease in the total extent of extensive livestock production systems in both developing and developed countries.
- Spatial movement (extensive livestock production will be practiced in areas and regions where it was impossible before). The flipside of this will be that extensive systems will disappear from areas where it was traditionally practiced.
- Camps/paddocks will have to be re-designed to allow for:
  - More shaded areas (trees or artificial).
  - More and strategically placed water points.
  - Smaller enclosed areas (camps/paddocks) to allow for less energy expenditure while grazing and visiting water points.
  - Strategically placed solar-powered lighting to enable animals to graze at night/cooler periods of the day and to rest during hotter periods of the day.
- Farming units will increase in size with less animals per area unit.
- Emphasis will shift to conservative stocking rates, pasture conservation, and rainwater harvesting.
- Indigenous/adaptive breeds will dominate but should not be to the detriment of production levels.
- Production efficiency will become paramount:
  - Survivability (disease, heat, and drought tolerance).
  - Reproduction efficiency/fertility.
  - Feed conversion rates.
  - Actual production (kilogram of meat per hectare) on natural or planted pasture utilized.
- Marker-assisted selection will become more relevant for the genetic improvement of extensive production animals.
- Diversification of species will be needed (mixture of small and large stock).
- Small stock species will begin to dominate over large stock species.
- Goats will become a species of choice in some areas due to their grazing/browsing capabilities.
- Pastoralism will come under pressure but might also provide solutions to climate change due to its adaptive nature.
- The production cost of extensive livestock farming will increase to some extent with subsequent increase in product price and potential consumer resistance.
- A relatively high skill set level will be required of extensive livestock farmers to deal with the adaptation/mitigation aspects of climate change.

## How Will Intensive Systems Adapt/Change under a Predicted Climate Change Scenario?

It is suggested that intensive livestock production systems will come under relatively less pressure compared with extensive systems. Intensive production systems may actually become the more favored choice. The following are predicted adaptive changes to be made to cope with a changed climatic scenario and to satisfy increased product demand:

- There will be an increase in intensive livestock production.
- Monogastric species will be seen as more “environmentally friendly” and will to some extent displace the current ruminant component.
- Intensive livestock production will move closer to the urban areas (urbanization of the production system) in the near future.
- Housing systems will change considerably with self-sufficient energy supply, air filtration, recycling of water, and sophisticated cooling systems.
- The spatial placement of housing systems will allow for smaller units with fewer animals per unit and be placed in such a way as to enhance biosecurity.
- Ruminant and monogastric diets will become more refined, keeping in mind the life cycle environmental production cost of the components used.
- Drought tolerant grains will form part of ruminant and monogastric diets as opposed to less drought-tolerant varieties.
- Manure management of intensive systems will become industrial processes to minimize environmental impact and to generate re-usable energy.
- Genetic selection will be leaning toward bigger fast growing animals which will be more efficient under intensive conditions.
- Marker-assisted selection will become essential for the genetic improvement of intensive production animals.
- There will be a shift from extensive to intensive production systems in developing countries.
- Developing countries will increase their share in the total production of animal protein as their resource base still lends itself for the expansion of animal production.
- The production cost of intensive livestock farming will increase considerably with subsequent increase in product price and potential consumer resistance.
- A “very high” skill set level will be required of intensive livestock farmers to deal with the adaptation/mitigation aspects of climate change.

## Conclusion

The bottom line is not to attempt to predict the future but rather to have all the relevant data available (both historic and modeled predictions) to make informed decisions. All relevant information should be used by animal scientists, veterinarians, climatologists, and farmers together with trends observed in practice to adjust a specific production system as the situation develops. If predictions are correct, climate change and the effects thereof will be a relatively slow process. It will, therefore, allow time for adjustments to be made to negate the effects of climate change. However, it is advisable not to delay these changes and rather implement them preemptively to buffer and negate the potential impact of climate change. It is, however, true to speculate that regardless if and to what extents climate change will occur, changes will have to be made to our current “way of doing things.” This is already demanded by the current and predicted increase in protein consumption with climate change having a confounding effect. These suggested changes will put us in a position to deal with climate more effectively since these adaptive changes also contain many mitigation elements which in turn will create a win-win situation for livestock production in its totality.

## About the Author

Dr Jean Rust is a senior scientist currently based at the Animal Research Directorate of the Döhne Agricultural Development Institute, Eastern Cape Province, South Africa. His postgraduate studies (MSc—University of Stellenbosch and PhD—University of the Free State, South Africa), focused on assisted reproduction in farm animals. He previously held a senior scientist/management position at the South African Agricultural Research Council. Dr Rust still practices as an assisted reproduction specialist, but his research interest has shifted to developing agriculture with a special focus on the development of alternative livestock production systems for commercial, smallholder, and subsistence farmers.
